# The Fascinating Effects of Baicalein on Cancer: A Review

**DOI:** 10.3390/ijms17101681

**Published:** 2016-10-09

**Authors:** Hui Liu, Yonghui Dong, Yutong Gao, Zhipeng Du, Yuting Wang, Peng Cheng, Anmin Chen, Hui Huang

**Affiliations:** 1 Department of Orthopedics, Tongji Hospital, Tongji Medical College, Huazhong University of Science and Technology, Wuhan 430030, China; liuhui09270808@gmail.com (H.L.); dongyonghuitjmu@gmail.com (Y.D.); gnituygnaw@163.com (Y.W.); pengcheng201507@163.com (P.C.); anminchen@hust.edu.cn (A.C.); 2 Institute of Pathology, Tongji Hospital, Tongji Medical College, Huazhong University of Science and Technology, Wuhan 430030, China; gaoyutong89@163.com; 3 Department of Gastroenterology, Tongji Hospital, Tongji Medical College, Huazhong University of Science and Technology, Wuhan 430030, China; herrdu@126.com

**Keywords:** baicalein, flavonoids, MAPK, Akt, reactive oxygen species (ROS), cancer, therapy

## Abstract

Cancer is one of the leading causes of death worldwide and a major global health problem. In recent decades, the rates of both mortality and morbidity of cancer have rapidly increased for a variety of reasons. Despite treatment options, there are serious side effects associated with chemotherapy drugs and multiple forms of drug resistance that significantly reduce their effects. There is an accumulating amount of evidence on the pharmacological activities of baicalein (e.g., anti-inflammatory, antioxidant, antiviral, and antitumor effects). Furthermore, there has been great progress in elucidating the target mechanisms and signaling pathways of baicalein’s anti-cancer potential. The anti-tumor functions of baicalein are mainly due to its capacities to inhibit complexes of cyclins to regulate the cell cycle, to scavenge oxidative radicals, to attenuate mitogen activated protein kinase (MAPK), protein kinase B (Akt) or mammalian target of rapamycin (mTOR) activities, to induce apoptosis by activating caspase-9/-3 and to inhibit tumorinvasion and metastasis by reducing the expression of matrix metalloproteinase-2/-9 (MMP-2/-9). In this review, we focused on the relevant biological mechanisms of baicalein involved in inhibiting various cancers, such as bladder cancer, breast cancer, and ovarian cancer. Moreover, we also summarized the specific mechanisms by which baicalein inhibited the growth of various tumors in vivo. Taken together, baicalein may be developed as a potential, novel anticancer drug to treat tumors.

## 1. Introduction

Cancer is one of the leading causes of death worldwide and a major global health problem [1]. In fact, mortality and morbidity rates are continuing to rise in both the developed and developing areas of the world. As such, there needs to be much more attention paid to this public health burden. In terms of childhood cancer specifically, the survival rate has dramatically increased over time in developed countries, while it remains low in low- and middle-income countries due to economic, genetic, and environmental factors [2]. With progress in science and technology, there have been unprecedented advances in the diagnosis and treatment of cancer. However, the majority of cancers still present an insurmountable challenge for the current medical system. Today, the primary treatment approaches for most tumors are surgery, radiotherapy, chemotherapy, and immunotherapy. Despite these options, treatment effects are significantly reduced because of the serious side effects of chemotherapy drugs and the occurrence of multiple forms of drug resistance.

Traditional Chinese medicine (TCM) has attracted considerable attention in recent years due to its effective treatment outcomes in relation to human diseases. For example, arsenic trioxide, a major component of arsenic, is an effective agent in treating patients with acute promyelocytic leukemia [3]. Artemisinin, one of the major compounds extracted from sweet wormwood, is a leading treatment for patients with malaria [4]. Vinca alkaloids, extracted from catharanthus roseus, have achieved great success in curing cancers [5]. Among multitudinous herbs, flavonoids are becoming more accepted as chemotherapeutic and dietary chemoprevention agents [6,7]. Furthermore, these natural agents have many advantages such as better accessibility and affordability, as well as lower toxicity in comparison with traditional chemotherapy agents [8]. Baicalein is a flavone and an active ingredient in the traditional herb, Huang Qin. There is an accumulating amount of evidence that demonstrates baicalein’s role in treating and preventing various types of cancer [9,10,11,12,13,14,15]. In this review, we explored its chemical structure, properties, and possible biological mechanisms through which it acts. Furthermore, our goal was to understand more fully the potential molecular mechanisms and targets of baicalein (both in vitro and in vivo) in the hope that this novel, anticancer agent could be used in future cancer treatments.

## 2. The Property and Antitumor Effect of Baicalein

**Figure 1:**
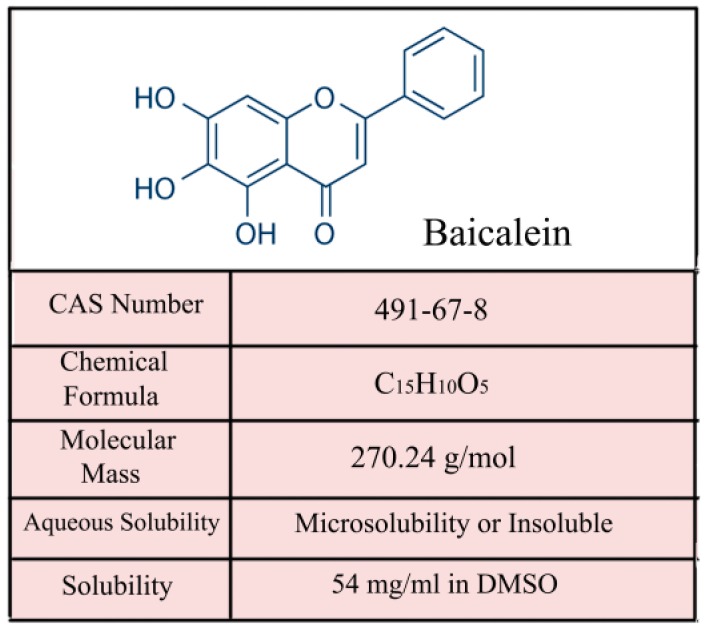
The chemical structure and major properties of baicaelin (5,6,7-trihydroxyflavone). CAS, chemical abstracts service; DMSO, dimethyl sulfoxide.

**Figure 2:**
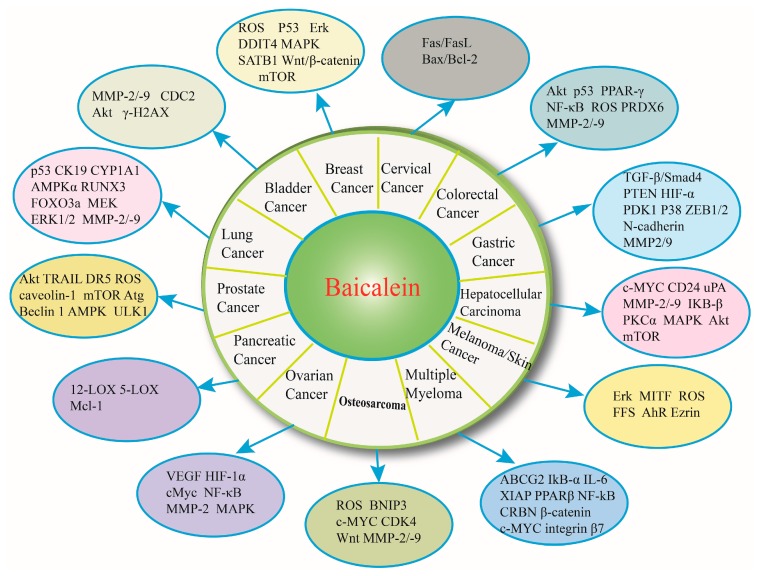
Baicalein inhibited various cancers through binding to and interacting with several molecular targets, such as Erk, ROS, MMP-2/-9, p53 and MAPK. These specific cellular targets which were involved in inhibiting various cancers are shown in Figure 2.

Baicalein is one of the major, active constituents of Scutellariae radix (also known as Chinese Huang Qin) and is isolated from its root. The prominent, structural feature of baicalein is the di-orthohydroxyl functional group, which is found on ring-A in its molecular structure [16]. The chemical structure and properties of baicalein are shown in Figure 1. Emerging evidence has demonstrated that baicalein exerts multiple pharmacological effects including anti-inflammatory [17], antioxidant [18], and antiviral [19] properties, as well as protection against cardiovascular illness [20]. In the past decade, there has been great progress in exploring the target mechanisms and signaling pathways of baicalein’s anti-cancer potential. The main molecular mechanisms of the anti-tumor effects of baicalein include inhibiting several cyclins or cyclin-dependent kinases (CDKs) to regulate the cell cycle [9], scavenging oxidative radicals, attenuating MAPK, Akt or mTOR activities [15], inducing apoptosis through activating caspase-9/-3 [11] and inhibiting tumor invasion and metastasis by reducing the expression of MMP-2/-9 [14]. As shown in Figure 2, we summarized the relevant biological mechanisms of baicalein involved in inhibiting various types of cancer (e.g., bladder cancer, breast cancer, cervical cancer, colorectal cancer, gastric cancer, hepatocellular carcinoma, osteosarcoma, multiple myeloma, melanoma/skin cancer, ovarian cancer, pancreatic cancer, prostate cancer, and lung cancer). This review attempts to elucidate the various antitumor effects of baicalein.

## 3. Baicalein and Bladder Cancer

Bladder cancer is one of the most frequent, primitive malignancies and the second most common cancer of the urinary system after prostate cancer [21]. According to the latest estimates in the USA, there will be 76,960 newly diagnosed cases of bladder cancer in 2016, and 16,390 deaths caused by bladder cancer [22]. Urothelial carcinoma is the most common, histological type of bladder cancer, accounting for about 90% of cases. The primary treatment options for patients are transurethral resections of their tumors and intravesical chemotherapy [23]. However, various side effects and resistance to chemotherapeutic agents are both barriers to the effectiveness of treatment. A recent study reported that baicalein induced apoptosis in T24 bladder cancer cells, by activating the mitochondrial-dependent caspase pathway and inhibiting Akt phosphorylation. More specifically, baicalein inhibited the growth of T24 cells by blocking cell cycle progression at the G1/S phase of division and induced apoptosis by activating caspase-9 and caspase-3, downregulating Bcl-2 expression, and upregulating Bax expression [24]. Results from another study demonstrated that baicalein inhibited the proliferation of human bladder papillary transitional carcinoma cells by arresting cell cycle progression in the G1 and S phase of division. More specifically, baicalein arrested cell cycle progression by decreasing the protein levels of cyclin B1 and cyclin D1. In addition, baicalein reduced the expression and activity of matrix metalloproteinase-2/-9, and subsequent invasion of cancer cells [9]. Some researchers have also reported that baicalein inhibited the growth of bladder cancer cells by inhibiting the activation of CDC2/cyclin B1 [25]. Baicalein also induced cell death by reducing the expression of securin, while also inhibiting cancer cell death by affecting the expression of p-AKT and γ-H2AX [26]. Furthermore, a study on in vivo tumor xenografts in C3H/HeN mice showed that baicalein significantly inhibited tumor growth [27]. Interestingly, another in vivo experiment revealed that baicalein did not affect the growth of bladder tumors [9]. The different results between these two animal models may be due to the different doses and dosing frequencies of baicalein. Collectively, these in vitro and in vivo studies suggested that there is great promise for treating bladder cancer with baicalein.

## 4. Baicalein and Breast Cancer

Breast cancer is one of the most frequent, primitive malignant tumors in women and has a high probability of metastases and poor prognosis. It is estimated that in the USA, there will be 246,660 new cases of breast cancer and 40,450 deaths as a result of breast cancer in 2016 [22]. A preliminary study attempted to investigate the molecular mechanism of baicalein-induced apoptosis in human breast cancer MD Anderson (MDA)-MB-231 cells. Baicalein (25–100 µM) disrupted the level of mitochondrial potential, released cytochrome c into the cytoplasm, and activated caspase-3. In addition, the anti-apoptotic protein Bcl-2 was downregulated, while the pro-apoptotic protein Bax was upregulated [28]. A study published in 2002 reported that baicalein strongly induced estrogen receptor (ER)-positive michigan cancer foundation-7 (MCF-7) cell apoptosis via suppressing 17β-estradiol-induced transactivation [29]. Another follow-up study indicated that baicalein-8-sodium sulfonate (BcS), a baicalein sulfated derivative, displayed a strong inhibition proliferation effect on MCF-7 cells via a ROS-dependent apoptosis pathway [30]. A combination study of baicalin and baicalein was performed showing their efficacy at causing apoptosis in MCF-7 and MDA-MB-231 cells. This study indicated that the combination of baicalin and baicalein had a synergistic effect on inducing apoptosis in tumor cells by activating caspase-3 and caspase-9, reducing Bcl-2 expression, and increasing Bax or p53 expression via the extracellular signal-regulated kinase (ERK)/p38 MAPK pathway [31]. Additionally, some studies reported that baicalein effectively suppressed adhesion, migration, invasion and metastasis in breast cancer cells. One study reported that baicalein inhibited cell adhesion and migration of MDA-MB-231 cells by reducing the expression of MMP-2/9 and downregulating MAPK [32]. Another study indicated that baicalein not only suppressed proliferation and invasion of MDA-MB-231 cells in vitro but also inhibited tumor metastasis in vivo. This study provided evidence for using baicalein as a promising agent in the treatment of metastasis in patients with breast cancer. The molecular mechanism of baicalein works via inhibition of epithelial-to-mesenchymal transition (EMT) and downregulation of special AT-rich sequence-binding protein-1 (SATB1) and Wnt/β-catenin pathways [10]. Consistent with the results of a former study by Ma et al., Gao et al. also reported that baicalein inhibited the proliferation, migration, and invasiveness of MDA-MB-231 cells by suppressing the expression of SATB1. SATB1 is highly expressed in numerous solid tumors and considered to be one of the important target molecules for antitumor agents [33]. Furthermore, a recent study reported that baicalein inhibited tumor growth in vivo via upregulation of DNA-damage-inducible transcript 4 (DDIT4) expression, which mediated the inhibition of mTOR in a breast cancer xenogeneic mouse model [34]. Collectively, these studies demonstrated that baicalein could be developed as a promising drug against breast cancer by targeting different pathways.

## 5. Baicalein and Cervical Cancer

A small number of publications have researched the potential of baicalein in treating patients with cervical cancer. According to estimates in the USA, there will be 12,990 new cases of cervical cancer and 4120 deaths related to this disease in 2016 [22]. Cervical cancer remains an important health concern for women because of its high prevalence and morbidity rates in developing countries. A recent study reported that baicalein induced apoptosis in human cervical cancer via the activation of the mitochondrial and death-receptor pathways. Results from this study showed that baicalein inhibited the proliferation of HeLa cells through upregulating the ratio of Bax/Bcl-2 expression and promoting the activation of Fas, FasL, and caspase-8 [11]. Another in vivo study revealed similar results in mice with U14 cervical cancer. This study showed that baicalein obviously inhibited tumor growth and induced apoptosis in U14 cervical cancer cells by upregulating the expression of Bax and downregulating the level of Bcl-2 [35]. These studies indicated that baicalein may be a promising agent for treating patients with cervical cancer.

## 6. Baicalein and Colorectal Cancer

Colorectal cancer (CRC) is the third most common malignant neoplasm. It has been a growing health problem worldwide and has seriously damaged the health of millions people [36]. In the USA, it is estimated that there will be 133,490 cases of newly diagnosed colorectal cancer, as well as 49,190 colorectal cancer-related deaths in 2016 [22]. Baicalein has been shown to induce DNA fragmentation and chromatin condensation, as well as block cell cycle progression in the G1 phase of division in HT-29 human colon cancer cells. In addition, the anti-apoptotic protein Bcl-2 was downregulated while the pro-apoptotic protein Bax was upregulated in a dose-dependent manner. The in vivo results of this study also proved that baicalein promoted apoptotic cell death of HT-29 colon tumor xenografts. These results also indicated that baicalein-induced apoptosis is mediated through Akt activation in a p53-dependent manner [37]. Baicalein treatment also arrested the cell cycle at the S phase of division and promoted apoptosis in human colorectal adenocarcinoma cells (HCT116 cells) via the activation of caspase-3 and -9 [38]. Another study reported that baicalein inhibited the growth of HCT116 cells and induced cellular apoptosis. Additionally, baicalein has the ability to exert chemopreventive and anti-inflammatory effects in the colon; this is related to inflammation associated-carcinogenesis through activating PPARγ and subsequent inhibition of NF-κB activation. This was an in vivo study that also supported the findings of an in vitro study revealing baicalein’s antitumor activity in azoxymethane/dextran sodium sulphate (AOM/DSS)-induced colitis and a colon cancer model in mice [39]. Both MMP-2 and MMP-9 are crucial proteases for the invasion and migration of CRC cells. Treatment of CRC cells with baicalein significantly inhibited cellular migration and invasion by suppressing the activities of MMP-2 and MMP-9 (via inhibition of the Akt pathway) [12]. Another group looked at the effect of baicalein on DLD-1 cells (colorectal adenocarcinoma cells) using proteomic analysis. Their results showed that baicalein was able to exert an inhibition effect on the proliferation of DLD-1 cells and decrease the level of reactive oxygen species (ROS) through up-regulating the expression of peroxiredoxin-6 (PRDX6) [40]. Collectively, these findings established that baicalein could be developed as a promising antitumor drug to treat colorectal cancer.

## 7. Baicalein and Gastric Cancer

Gastric cancer (GC) is the fourth most common aggressive cancer in the world. There are approximately 990,000 newly-diagnosed cases and about 740,000 gastric cancer-related deaths every year [41]. The main treatment strategy for patients with gastric cancer remains a combination of surgery, chemotherapy, and radiotherapy. However, the prognosis of these patients is still poor due to the various side effects and multiple forms of drug resistance. A recent study reported that baicalein induced cell cycle arrest at the S phase of division and promoted apoptosis in SGC-7901 gastric cancer cell lines via downregulation and upregulation of Bcl-2 and Bax levels, respectively. An in vivo study also revealed that baicalein excellently inhibited tumor growth in subcutaneous xenograft models [42]. Baicalein treatment inhibited the migration and invasion of GC cells in Transwell and wound-healing assays. Baicalein also decreased the expression of metastasis-associated molecules, including *N*-cadherin, vimentin, ZEB1, and ZEB2. This study provided the basic mechanism for baicalein’s inhibition of metastasis in gastric cancer cells by inactivation of the TGF-β/Smad4 pathway [43]. Similarly, another study revealed that baicalein inhibited the invasion of gastric cancer cells by inhibition of the p38 signaling pathway and subsequent MMP-2 and MMP-9 suppression [44]. Baicalein was reported to reverse hypoxia-induced 5-FU resistance in gastric cancer AGS cells. Baicalein enhanced the sensitivity of AGS cells to 5-FU and suppressed glycolytic flux. Furthermore, it suppressed the expression of glycolysis-associated enzymes such as HK2, LDH-A, and PDK1 under hypoxic conditions, possibly via activation of the PTEN/Akt/HIF-1α pathway [45]. In conclusion, these studies reveal that baicalein may be developed as an effective therapy for treating patients with gastric cancer.

## 8. Baicalein and Hepatocelluar Carcinoma

Hepatocelluar carcinoma (HCC) is the fifth most common cancer worldwide. It was also the third-leading cause of cancer-related deaths in 2015 [46]. Most cases of hepatocellular carcinoma occur in underdeveloped areas of the world (especially those found in Asia and Africa). China has the highest incidence of HCC of any country and, as such, is one of the main reasons for its high number of patients with chronic hepatitis B. In recent decades, the incidence of HCC has gradually risen in the developed world (e.g., North America and Europe) [13]. Hence, the treatment of HCC with baicalein has attracted additional attention in the field of cancer research. Cell viability assays have demonstrated that baicalein is significantly cytotoxic against HCC cells. Baicalein induced both apoptosis and cell cycle arrest in the G0/G1 phase of division in HCC cells, by modulating the transcription of cyclin D1 through a β-catenin-dependent mechanism [13]. Another study reported that baicalein inhibited tumor cell proliferation and induced G2/M arrest in HCC J5 cells. In addition, baicalein stimulated apoptosis via a mitochondrial-dependent caspase pathway, which included the promotion of the release of mitochondrial cytochrome c, apoptosis inducing factor (AIF) and Endo G, activating caspase-9 and -3, and increasing the ratio of Bax/Bcl-2 [47]. Chang et al. also confirmed that baicalein induced Hep G2 cell apoptosis by mitochondrial dysfunction. A recent study demonstrated that baicalein suppressed the expression of c-Myc, a key regulator of cell proliferation and apoptosis in HCC cells. Moreover, the expression of CD24 plays a role in the growth of HCC and was downregulated by baicalein. Therefore, this study implied that baicalein inhibited the growth and survival of HCC cells via downregulation of c-Myc and CD24 levels [48]. Furthermore, HQS-3, a new derivative of baicalein, distinctly inhibited the growth of transplantable Heps tumors in mice and xenograft tumors in nude mice. The treatment of HepG2 cells with HQS-3 induced mitochondrial-mediated apoptosis by generating ROS and downregulating the level of the anti-oxidative protein SOD2 [49]. Some researchers demonstrated that baiclalein inhibited cellular adhesion, migration, invasion, and growth of HCC cells both in vitro and in vivo. Their findings showed that baicalein inhibited cell-ECM interactions by suppressing the activities of MMP-2, MMP-9, and u-PA. In addition, the nuclear translocation of p50 and p65, and the phosphorylation of I-kappa-B (IΚB)-β, were also downregulated by baicalein. This study provided a molecular mechanism for using baicalein as an anti-metastatic agent by inhibiting the expression of protein kinase C α (PKCα) and p38 MAPK [50]. The latest study found that baicalein triggered protective autophagy in HCC cells. The AKT/mTOR pathway is known as the key regulator of autophagy and was inhibited by baicalein in HepG2 cells [51]. Another study showed that baicalein induced autophagy via endoplasmic reticulum stress in human HCC cell lines SMMC-7721 and Bel-7402 [52]. These findings determined that the combination treatment of baicalein and autophagy inhibitors may be more effective as an anticancer therapy. In addition, the treatment of hepatoma HepG2 cells with a combination of baicalein and silymarin was obviously superior to baicalein or silymarin alone [53]. This combined treatment provided a novel insight for future clinical therapy of HCC. Together, this information suggests that baicalein is a promising candidate for the treatment of HCC.

## 9. Baicalein and Melanoma/Skin Cancer

Melanoma is a potentially malignant and lethal type of skin cancer. According to the latest estimates in the USA, there will be 76,380 new cases of melanoma and 10,130 melanoma-related deaths in 2016 [22]. Therefore, more in-depth research is required to treat this disease. Some early studies found that baicalein inhibited the growth of B16F10 melanoma cells [54]. A recent study also reported that baicalein inhibited melanogensis in B16F10 mouse melanoma cells by activating the ERK signaling pathway. Furthermore, in vitro studies revealed that the reduction of tyrosinase activity, downregulation of microphthalmia-associated transcription factor (MITF) protein expression, and activation of ERK have been involved in the mechanisms of baicalein-mediated inhibition of melanogensis [55]. Another study showed that baicalein inhibited the proliferation of B16F10 melanoma cells through catalyzing the formation of reactive oxygen species (ROS) by 12-lipoxygenase [56]. A follow-up study revealed that co-treatment of melanoma with γ- tocotorienol and baicalein had a synergistic inhibitory effect on its growth. This effect was exerted by modulating the levels of food factor sensing (FFS), especially the level of aryl hydrocarbon receptor expression (AhR) [57]. In addition to this research on melanoma, a non-melanoma skin cancer study reported that baicalein significantly inhibited 7,12-Dimethylbenz[a]anthracene (DMBA)/12-*O*-Tetradecanoylphorbol-13-acetate (TPA) induced skin tumorigenesis. In fact, baicalein treatment inhibited cell proliferation and induced apoptosis. Furthermore, baicalein suppressed inflammation in DMBA/TPA induced skin cancer and reduced skin hyperplasia, as well as leukocyte infiltration [58]. Ezrin is highly expressed in skin cancer, plays an important role in the metastasis of tumors, and was found to be involved in the inhibition of skin carcinoma by baicalein. Specifically, baicalein (2.5–40 μM) inhibited the migration and invasion of A431 skin carcinoma cells via suppression of Ezrin and phos-Ezrin expression [59]. Collectively, these studies demonstrate that baicalein could be developed to treat patients with skin cancer.

## 10. Baicalein and Multiple Myeloma

Multiple Myeloma (MM) is one of the most common incurable plasma-cell malignancies and the second most frequent hematologic malignancy. It is characterized by extensive bone destruction, a disposition to repeated infection, hyperviscosity syndrome, renal insufficiency, etc. [60]. In the USA, it is estimated that there will be 30,330 new cases of MM and 12,650 MM-related deaths in 2016. As such, MM remains a severe threat to people’s health [22]. A study involving Scutellaria Extract was conducted to examine its effects on the human multiple myeloma cell line, RPMI8226. This study found that baicalein, the major component in Scutellaria Extract, significantly reduced the number of side population (SP) cells of RPMI8226 by decreasing the expression level of ABCG2 protein [61]. Another study reported that baicalein inhibited the cell viability of RPMI8226 cells (IC50 value of 168.5 μM) and also reduced the proportion of SP cells by inhibiting the expression of ABCG2 [62]. Huang-Lian-Jie-Du-Tang (HLJDT) is a traditional Chinese Herbal medicine. Ma et al. found that baicalein, one of HLJDT’s main and active components, strongly inhibited the proliferation and survival of either primary myeloma cells or myeloma cell lines [63]. Furthermore, baicalein induced apoptosis of myeloma cell lines by inhibiting the phosphorylation of IκB-α and reducing the expression of the IL-6 and XIAP genes. This subsequently caused a change in mitochondrialmembrane potential and the activation of caspase-9 and -3. IL-6 has been identified as a promoter for the survival and proliferation of MM cells. Baicalein could be developed as a potent inhibitor of protein phosphorylation (induced by IL-6) to treat patients with MM [64]. In addition, the combination treatment of baicalein with dexamethasone exerted the prominent suppression of MPC-1-immature myeloma cells by activating both PPARβ and glucocorticoid receptors (GR). This had a synergistic, inhibitory effect on the transcriptional activity of nuclear NF-κB [65]. Baicalein can induce the expression of cereblon (CRBN) and cause MM cells to overcome their resistance to lenalidomide. Therefore, it was confirmed that combination therapy (baicalein and lenalidomide) was more effective than monotherapy [66]. Baicalein treatment also inhibited the proliferation and migration of MM cells by downregulating the expression level of β-catenin, c-myc, cyclinD1, and integrin β7 [67]. Thus, baicalein could hold great promise as a treatment for patients with MM. However, additional research needs to be conducted before advancing to clinical application.

## 11. Baicalein and Osteosarcoma

Osteosarcoma is the most common malignant bone tumor worldwide, especially in adolescents and young adults. Osteosarcoma occurs principally in the metaphysis of long bones, including the distal femur, proximal tibia, and proximal humerus [68]. Currently, the primary treatment for osteosarcoma is wide surgical removal of the tumor and intensive adjuvant chemotherapy. However, there has been no significant improvement in the 5-year survival rate since the 1970s and the outcomes of available treatments remain unsatisfactory [69]. A recent study demonstrated that baicalein inhibited proliferation and promoted apoptosis of MG-63 osteosarcoma cells via intracellular ROS generation and activation of BNIP3 [70]. Another study showed that baicalein induced apoptosis of MG-63 cells by targeting the c-MYC gene and activating the Wnt signaling pathway [71]. In addition, Zhang et al. found that baicalein inhibited osteosarcoma cell growth by reducing the expression of cyclin D1 and cyclin-dependent kinase 4 (CDK4), followed by subsequent blocking of cell cycle progression at the G1 phase of division. Baicalein treatment also induced apoptosis by activating caspase-3, downregualtingBcl-2 expression, and upregulating Bax expression. Furthermore, baicalein decreased both the migration and invasion of osteosarcoma cells by reducing the expression of MMP-9 and MMP-2 [72]. HSP70, which is essential for the survival and partial protection of tumor cells from apoptosis, was upregulated after baicalein treatment. This finding suggested that the anti-osteosarcoma property of baicalein can be strengthened in combination with some inhibitors of HSP70 [73]. Broadly speaking, baicalein could be a potential future treatment of patients with osteosarcoma.

## 12. Baicalein and Ovarian Cancer

Ovarian cancer is unique to the female population and is a deadly disease worldwide, with a 5-year survival rate of only 46.2%. According to the latest forecast in the USA, there will be 22,280 newly diagnosed cases of ovarian cancer and 14,240 ovarian cancer-related deaths in 2016 [22]. Most patients with ovarian cancer are usually diagnosed at advanced stages, by which time the tumor cells have metastasized to either the peritoneal or pelvic cavities. Vascular endothelial growth factor (VEGF), a key regulator of vasculogenesis, plays an important role in both tumor growth and metastasis [74]. He et al. found that baicalein exerted an inhibitory effect on the expression of VEGF in human ovarian cancer cell lines [75]. Another study further concluded that baicalein inhibited ovarian cancer cell viability to a greater extent than baicalin. In addition, baicalein suppressed the expression of VEGF, HIF-1α, c-Myc, and NF-κB in ovarian cancer cell lines (OVCAR-3 and CP-70). However, baicalein had fewer effects on the viability of normal ovarian cell lines (IOSE-364) [76]. Recent research has demonstrated that baicalein inhibited the invasion of ovarian cancer cells by decreasing the expression level of MMP-2. Baicalein also suppressed the activation of a p-38 MAPK-dependent NF-κB signaling pathway [77]. Therefore, baicalein could be an effective treatment against ovarian cancer.

## 13. Baicalein and Pancreatic Cancer

Pancreatic cancer (PaCa) is the fourth leading cause of cancer-related death in the USA [78] and has a 5-year survival rate of less than 5% [79]. It is known to be an enigmatic and aggressive malignancy with an extremely high mortality rate. In the USA, it is estimated that there will be 53,070 new cases of PaCa in 2016, with 41,780 PaCa-related deaths [22]. Early surgical resection is the only treatment that cures PaCa, which is only applied to a small portion of patients. Therefore, we are looking for new agents to fight this disease. Ding et al. found that both 5-lipoxygenase (5-LOX) and 12-lipoxygenase (12-LOX) were upregulated in PaCa cells and that inhibition activation of these enzymes suppressed cellular growth [80]. Baicalein is an inhibitor of 12-LOX and induced apoptosis, morphological changes, and carbonic anhydrase expression in PaCa cells. Baicalein also promoted mitochondrial cytochrome c release from mitochondria, increased the ratio of Bax/Bcl-2 expression, and activated caspase-9, caspase-7 and caspase-3. An in vivo study also confirmed that baicalein inhibited tumor growth in human pancreatic cancer cell (HPAC) or AsPC-1 athymic mice xenograft models [81]. Mcl-1 is a member of the Bcl-2 anti-apoptotic protein family and was downregulated in PaCa cells after treatment with baicalein. Furthermore, overexpression of Mcl-1 attenuated the apoptotic effect that was mediated by baicalein. Thus, baicalein induced the apoptosis of PaCa cells, in part, by downregulating the expression of Mcl-1 [82]. These findings suggest that baicalein may be developed into a promising therapeutic drug for patients with PaCa.

## 14. Baicalein and Prostate Cancer

Prostate cancer is a type of fatal genitourinary cancer and is one of the most frequent malignancies in men. It is also the second most common cancer in the USA, with an expected 180,890 newly diagnosed cases and 26,120 related deaths in 2016 [22]. Available treatments for prostate cancer include prostatectomy, radiation therapy, and androgen deprivation therapy. Despite these options, treatment outcomes remain unsatisfactory due to various side effects and resistance to castration. Baicalein has been studied in prostate cancer for many years. In one study, baicalein suppressed the growth of human prostate DU-145 and PC-3 cell lines in a dose-dependent manner in vitro. Furthermore, baicalein significantly reduced the volume of DU-145 tumor cells in severe combined immunodeficient mice (SCID) mice in vivo [83]. In addition, baicalein inhibited the growth of PC-3 cells by causing cell cycle arrest at the G0/G1 phase of division; this inhibition was associated with the suppression of both cyclin D1 and D3 expression. Baicalein also induced apoptosis by activation of both caspase-3 and -7, dephosphorylation of Akt, loss of survivin, and increasing the ratio of Bax/Bcl-2 expression in prostate cells [84]. TNF-related apoptosis inducing ligand (TRAIL) is a promising new candidate for cancer therapy as it plays a vital role in fighting tumor cells. However, some studies showed that certain cancers have a resistance to TRAIL. Baicalein lessened this resistance to TRAIL by upregulating DR5 expression and promoting the expression of ROS, thus causing TRAIL sensitization in PC3 cells [85]. Another study demonstrated that baicalein inhibited the caveolin-1/AKT/mTOR pathway in androgen-independent PCa cells, effectively augmenting apoptosis and weakening metastasis [15]. Furthermore, baicalein increased the sensitivity of prostate cancer cells to radiation without affecting this sensitivity in normal cells [86]. Interestingly, a recent study reported that baicalein resulted in human cancer cell death by inducing autophagy rather than apoptosis. This study found that baicalein-induced cell death could be reversed by inhibiting the expression of Beclin 1, vacuolar protein sorting34 (Vps34), autophagy-related (Atg) 5, and Atg7 (all important modulators in autophagy) instead of by a pan-caspase inhibitor. They also demonstrated that baicalein induced autophagic cell death in prostate cancer cells by activating the AMPK/ULK1 pathway and inhibiting the expression of anti-autophagic molecules of the mTOR/Raptor complex 1 [87]. These findings confirm that baicalein could be a promising agent for treating patients with prostate cancer.

## 15. Baicalein and Lung Cancer

Lung cancer remains one of the most common malignancies worldwide, with high rates of both incidence and mortality. In the USA alone, it is estimated that there will be 224,390 new cases of lung cancer and 158,080 lung cancer-related deaths in 2016 [22]. Worldwide, there are approximately 1.8 and 1.6 million newly diagnosed cases and deaths every year, respectively [88]. To find more effective adjuvant agents for the treatment of lung cancer, Gao et al. investigated a traditional Chinese herbal medicine known as Scutellaria baicalensis. Specifically, they researched the effects of S. baicalensis on both cell growth and apoptosis of human lung cancer cell lines A549, SK-LU-1, and SK-MES-1. They found that the ethanolic extracts of S. baicalensis inhibited proliferation of both A549 and SKMES-1 cells. The effect on A549 cells was accomplished by blocking the cell cycle in the S phase of division through decreasing the expression of cyclin A, whereas the effect on SKMES-1 cells was accomplished by blocking the cell cycle in the G0/G1 phase of division through decreasing the expression of cyclin D1. In addition, this herb enhanced apoptosis of lung cancer cells by increasing the expression of both p53 and Bax [89]. Another study reported that baicalein (IC50: 80 ± 6 uM), as one of the main components in S. baicalensis, played a major role in anti-proliferation effects [90]. Baicalein was shown to inhibit growth and induce apoptosis in non-small cell (NSC) lung cancer H460 cells. Its effects were exerted through the reduction of both cdk1 and cyclinB1 and upregulation of the Bax/Bcl-2 ratio, which led to arrest in the S-phase of division as well as activation of caspase-3 [91]. Similar results were also found in a study of human lung squamous carcinoma CH27 cells [92]. Baicalein (12 mg/kg body weight) treatment significantly decreased tumor incidence, arrested tumor multiplicity, and reduced tumor load. The serum tumor markers, such as Carcinoembryonic antigen, constitute a set of glycoproteins (e.g., CK 19 fragments and lactate dehydrogenase) that further substantiate the anticancer effects of baicalein in vivo [93]. In one study on sub-cellular activities, baicalein reversed damage to nuclear DNA and restored mitochondria that had suffered oxidative damage in Swiss albino mice with lung carcinogenesis [94]. Treatment with baicalein also inhibited lung carcinogenesis by counteracting lysosomal and microsomal abnormalities through the reduction of oxidative damages and downregulation of CYP1A1 [95]. Moreover, baicalein treatment inhibited proliferation and induced apoptosis of NSC lung cancer cells by phosphorylation of AMPKα and MEK/ERK1/2, and upregulating the expression of RUNX3 and FOXO3a [96]. Interestingly, baicalein effectively decreased the levels of TNF-α, IL-β, iNOS, COX-2, MMP-2, and MMP-9. As a result, inflammation associated with pulmonary carcinogenesis was inhibited, which helped to combat lung cancer [14]. Combination therapy of baicalein with paclitaxel, which were assembled by nanoparticles, was demonstrated to have synergistic anticancer effects in A549 lung cancer cells and in mice bearing A549/PTX drug-resistant lung cancer xenografts [97]. Collectively, baicalein showed extremely promising results in treating patients with lung cancer.

## 16. Potential Clinical Implication and Discussion

Multitudinous preclinical studies have proved that baicalein could be developed as a tremendous potential antitumor drug against cancer by targeting multiple molecular mechanisms and signaling pathways. In addition, accumulating evidence from animal studies demonstrates that baicalein significantly inhibited tumor volume and tumor weight, such as in hepatocellular cancer [98,99] and lung cancer [95]. Furthermore, it is worth noting that the development of appropriate delivery systems and chemical modification of baicalein can enhance its efficacy in preclinical studies. Unfortunately, very few clinical trials of baicalein were conducted to study its efficacy for the treatment of tumors in clinics. In order to promote the anti-tumor potential of baicalein to clinical use, in addition to some conventional animal experimental data, some issues, such as bioavailability and toxicological profiles of baicalein, have to be comprehensively investigated before clinical trials are initiated. In some tumors, such as breast cancer [34], colorectal cancer [37] and hepatocellular cancer [13], animal experiments suggested that baicalein significantly decreased tumor weights and volumes without toxicity. Therefore, it is of great significance to select some tumors such as breast cancer or coloreactal cancer to initiate the phase I and II trials.

## 17. Conclusions and Future Perspectives

As is well known, cancer is a multifactorial disease. In recent decades, the mortality and morbidity rates of cancer have rapidly increased due to environmental pollution, various carcinogens, unhealthy lifestyles, high stress levels, and pressures of work. Chemotherapy plays a vital role in the treatment of cancer patients by killing tumor cells. However, chemotherapy agents can also cause toxicity to surrounding normal tissue cells, resulting in a variety of side effects. Moreover, the effectiveness of chemotherapy is seriously limited by the occurrence of resistance. Therefore, finding a novel drug with few side effects and high efficacy has become an urgent trend in cancer therapy. Baicalein has garnered increasing attention in recent years due to its strong antitumor activity and low toxicity. In this review, we introduced various molecular mechanisms and signaling pathways to elucidate its anticancer potential, including blockage of the cell cycle, inducing apoptosis, inhibiting tumor cell invasion and metastasis, potentiating the actions of chemotherapeutic agents, triggering autophagic cell death, and so on. Moreover, we also summarized specific mechanisms by which baicalein inhibited various tumor growths in vivo in Table 1. It is worth noting that the bioavailability of baicalein in vivo remains low. Despite the promising results of preclinical studies on compounds derived from baicalein, additional research is required to improve their biological effects on various cancers. Nevertheless, based on the great number of studies, we have reason to believe that baicalein may potentially be developed as a novel anticancer drug, to be administered alone or applied with current popular chemotherapeutic drugs, thus improving the future treatment of cancer.

## Figures and Tables

**Table 1:** Summary of baicalein and its anti-tumor properties in vivo.

Cancer	Animal Models	Baicalein Dose	Conclusions	Reference
Bladder cancer	MBT-2 cell xenografts in C3H/HeN mice	0.05 and 0.1 mg/animal, i.h. for 10 days	Baicalein significantly inhibited the tumor growth	[27]
	MB49 cell xenograft in C57BL/6 mice	0.8 mg/animal, i.h. for 9 times	Baicalein slightly inhibited tumor growth with some hepatotoxicity	[9]
Breast cancer	MDA-MB-231 cell xenograft in nude mouse	50 or 100 mg/kg, b.wt., i.g. for 15 days	Baicalein suppresses breast cancer metastasis by inhibition of EMT via downregulation of SATB1 and Wnt/β-catenin pathway	[10]
	MDA468 cell xenograftin SCID-Bg mice	20 mg/kg, b.wt., i.p. for 5 days/week	Baicalein suppressed tumor growth of MDA468 cancer cells without toxicity to the host and increased DDIT4	[34]
Colorectal cancer	AOM/DSS-induced colon cancer	1, 5, 10 mg/kg, b.wt., orally for 16 weeks	Baicalein significantly decreased the incidence of tumor formation with inflammation	[39]
	HCT-116 cell xenograft in athymic nude mice	30 mg/kg, b.wt., i.p. every other day for 4 weeks	Baicalein showed more significant inhibition of tumor growth than those of its parent compound baicalin	[38]
	HT-29 cells xenografts in nude mice	10 mg/kg, b.wt, orally three times every week for 43 days	Baicalein significantly decreased tumor weights and volumes without toxicity	[37]
Gastric cancer	SGC-7901cell xenograft in nude mice	15 and 50 mg/kg, b.wt, i.g. for 1 week	Baicalein potently inhibited the weight and size of tumors	[42]
Hepatocellular cancer	H22 cell xenograft in ICR mice	50 and 100 mg/kg, i.p. for 13 days	Baicalein significantly inhibited the tumor growth without causing obvious adverse effects on weight or liver and spleen weight	[13]
	SK-Hep1cell xenograft in athymic BALB/c-nu mice	5, 10, 20 mg/kg/day; i.p. for 32 days	Baicalein was found to significantly decrease the solid tumor mass and reduced the number of PKCα-positive cells	[50]
	DEN-induced rat model	250 mg/kg, b.wt., i.g. for 2 weeks	Baicalein also reduced neoplastic nodules by inhibition of 12-LOX	[98]
	HepG2cell xenograft in nude mice	20 mg/kg/day, orally	Baicalein suppresses HCC xenograft growth via inhibition of MEK-ERK signaling and by inducing intrinsic apoptosis	[99]
Lung cancer	B(a)P-induced lung cancer	12 mg/kg, b.wt., orally for 16 weeks	Baicalein abrogates reactive oxygen species (ROS)-mediated mitochondrial dysfunction during experimental pulmonary carcinogenesis in vivo	[94]
	B(a)P-induced lung cancer	12 mg/kg, b.wt., orally for 16 weeks	Baicalein inhibited pulmonary carcinogenesis-associated inflammation and interfered with COX-2, MMP-2 and MMP-9 expressions in vivo	[14]
	B(a)P-induced lung cancer	12 mg/kg, b.wt., orally for 16 weeks	Baicalein effectively negated B(a)P-induced upregulated expression of CYP1A1 and inhibited lysosomal and microsomal dysfunction.	[95]
	B(a)P-induced lung cancer	12 mg/kg, b.wt., orally for 16 weeks	Baicalein significantly inhibited pulmonary adenoma formation and growth	[93]
Skin cancer	DMBA/TPA-induced skin tumor	25 mg/kg, b.wt. for 30 weeks	Baicalein inhibited DMBA/TPA-induced skin tumorigenesis in mice by modulating proliferation, apoptosis, and inflammation	[58]
	B[a]P/TPA-induced skin tumor	0.08, 0.16, or 0.2 pmol/animal; topical application	Baicalein inhibited the number of TPA-induced tumors per mouse significantly	[100]
Pancreatic cancer	HPAC and AsPC-1 cell xenograft in athymic mice	250 mg/kg/day b.wt., i.g. for 4 weeks	Baicalein greatly inhibited tumor volume and tumor weight	[81]
Prostate cancer	LNCaPcell xenograft in athymic mice	20 mg/kg/day, b.wt., p.o. for 4 weeks	Baicalein reduced the growth of prostate cancer xenografts in nude mice by 55% at 2 weeks through inhibition of the androgen receptor signaling pathway	[101]
	DU-145cell xenograft in SCID mice	10, 20, 40 mg/kg p.o. for 4 weeks	Treatment of mice with baicalein demonstrated a statistically significant tumor volume reduction	[83]

i.h: subcutaneous injection; b.wt: body weight; i.g: gavage; i.p: intraperitoneally; p.o: oral administration.
